# Vancomycin-sensitive bacteria trigger development of colitis-associated colon cancer by attracting neutrophils

**DOI:** 10.1038/srep23920

**Published:** 2016-04-06

**Authors:** Yuriko Tanaka, Sachiko Ito, Ken-ichi Isobe

**Affiliations:** 1 Department of Immunology, Nagoya University Graduate School of Medicine, 65 Tsurumai-cho, Showa-ku, Nagoya, Aichi, 466-8550, Japan; 2 Department of Food Science and Nutrition, Nagoya Women's University, 3-40 Shioji-cho, Mizuho-ku, Nagoya, Aichi, 467-8610, Japan

## Abstract

Inflammatory bowel disease confers an increased risk of developing colitis-associated colon cancer (CAC). During the active colitis or developing tumor stage, commensal bacteria show dynamic translocation. However, whether alteration of the bacterial composition in the gut causes CAC is still unclear. To clarify the effect of commensal bacteria on CAC development, we employed an azoxymethane (AOM) and dextran sodium sulfate (DSS)-induced murine CAC model treated with or without antibiotics. In addition, we analyzed the effects of antibiotics on infiltration of myeloid cells, colonic inflammatory responses, and colorectal cancer formation. We found that vancomycin treatment dramatically suppressed tumor development. In addition, AOM/DSS treatment greatly induced the infiltration of Gr-1^high^/CD11b^high^ neutrophils to the colon, which led to the production of tumor necrosis factor α and inducible nitric oxide synthase. Vancomycin treatment suppressed the infiltration of neutrophils induced by AOM/DSS. Moreover, vancomycin treatment greatly reduced the colon injury and DNA damage caused by AOM/DSS-induced NO radicals. Our results indicate that vancomycin-sensitive bacteria induced colon inflammation and DNA damage by attracting neutrophils into damaged colon tissue, thus promoting tumor formation.

Colorectal cancer is one of the most frequent human neoplasias and the third-highest cause of cancer deaths in industrialized countries^1^. Carcinogens cause mutations in oncogenes (K-ras, c-myc, c-src, c-neu) or tumor suppressor genes (p53, APC, Smad4) in colonic epithelial cells^2^. In addition to genetic abnormalities, the formation of an inflammatory microenvironment also plays a pivotal role in colorectal cancer development^3^. Chronic inflammation, such as that present in ulcerative colitis (UC) and Crohn’s disease, is associated with an increased risk of colorectal cancer^4^,^5^,^6^,^7^,^8^. The duration and severity of UC correlate with the risk of developing colitis-associated colon cancer (CAC)^9^,^10^,^11^.

In the human gut, there are approximately 10^13^ commensal bacteria dominated by *Bacteroidetes, Firmicutes*, and *Proteobacteria*^12^. Chronic inflammatory diseases of the gut are initiated by the aberrant interaction of the host immune system with commensal microflora^13^,^14^. Despite many studies of colitis in germ-free animals or animals treated with antibiotics, the contribution of commensal bacteria to colitis and colitis-associated carcinogenesis remains unclear. For example, some data indicate that antibiotics or a germ-free state can block the development of colitis^15^,^16^,^17^. In contrast, other evidence suggests that germ-free mice, and mice lacking individual toll-like receptors (TLR) or the critical TLR-signaling molecule MyD88 are more susceptible to dextran sodium sulfate (DSS)-induced colitis^18^,^19^,^20^,^21^.

Neutrophils and macrophages may play important roles in disease progression and/or host defense. Both neutrophils and macrophages belong to the myeloid cell lineage and multiply and differentiate in the bone marrow. We have previously shown that Gr-1^high^/CD11b^high^ neutrophils were enhanced in colon, bone marrow, and spleen in a DSS-induced murine colitis model^22^,^23^. However, whether the commensal bacterial composition affects the induction of Gr-1^high^/CD11b^high^ neutrophils in the colon, bone marrow, and spleen is still unclear.

Azoxymethane (AOM)/DSS models have been widely used to study inflammation-dependent colorectal cancer^24^,^25^. Here, we used this model in mice to examine the effects of commensal bacteria on colon inflammation and colorectal cancer. We showed that vancomycin suppressed colon carcinogenesis by inhibiting inflammatory responses and colon tissue damage.

## Results

### Vancomycin reduced the risk of colitis-associated carcinogenesis

**Figure 1:**
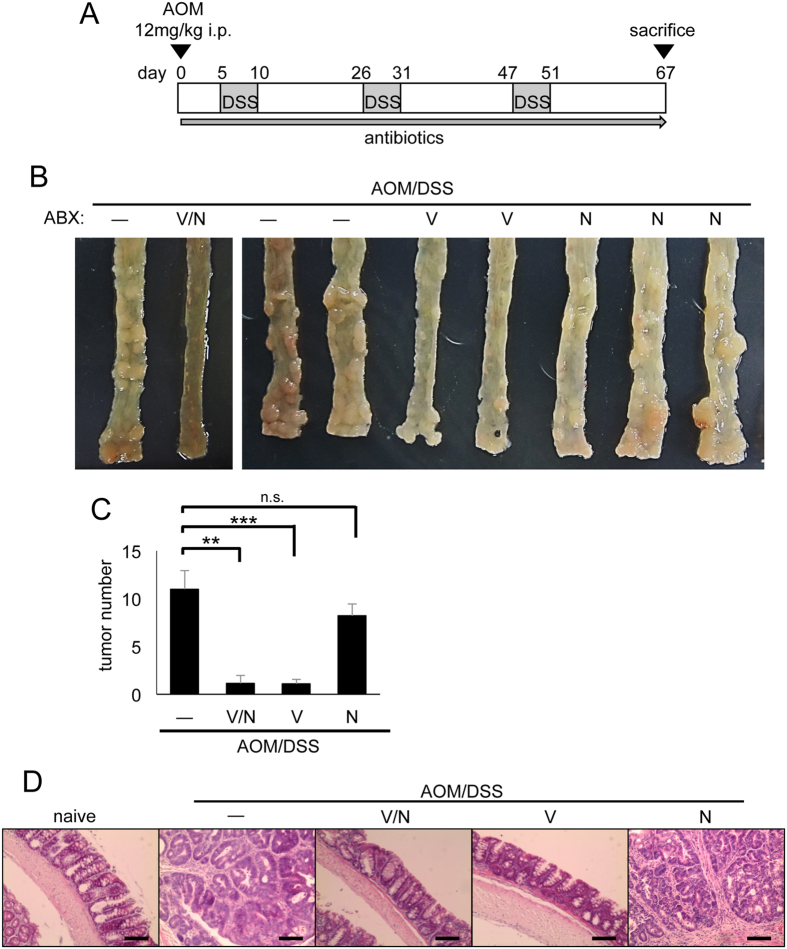
Vancomycin reduced the risk of colitis-associated carcinogenesis. (**A**) Schematic of mice treated with AOM/DSS. After initial AOM injection (12 mg/kg), DSS was given in drinking water followed by regular water three times. Antibiotics (combination of vancomycin and neomycin, vancomycin alone, or neomycin alone) were administered throughout this procedure. (**B**) Macroscopic view of tumor formation in colon-rectum regions of AOM/DSS-treated control mice and antibiotics (vancomycin/neomycin, vancomycin, or neomycin) -treated mice at day 67. (**C**) Statistical analysis of tumor number in colon-rectum regions at day 67. Data represent mean ± SEM (−, n = 8; V/N, n = 7; V, n = 8; N, n = 5). **p < 0.01, ***p < 0.001. (**D**) H&E staining of distal colon at day 67. Scale bar: 100 μm. ABX, antibiotics; AOM, azoxymethane; DSS, dextran sodium sulfate; H&E, hematoxylin and eosin; i.p., intraperitoneal; N, neomycin; n.s., not significant; SEM, standard error of the mean; V, vancomycin; V/N, vancomycin and neomycin.

**Figure 2:**
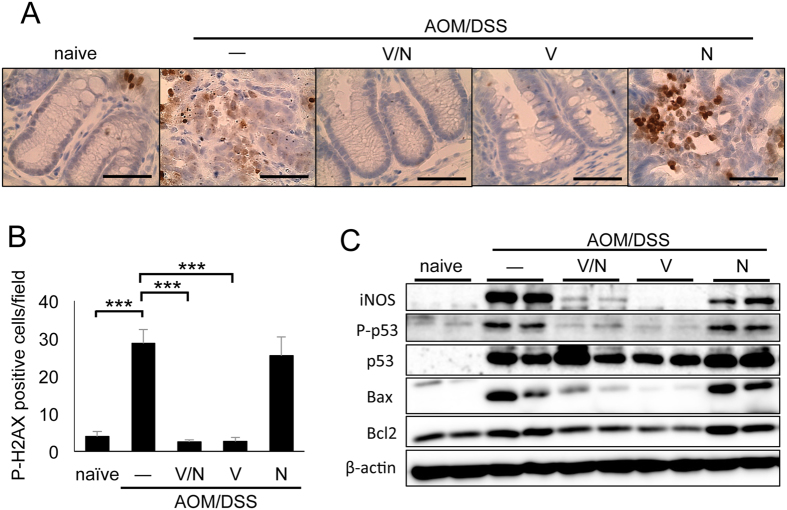
Vancomycin reduced DNA damage and cell death of colon epithelial cells. (**A**) Immunohistochemical analysis of distal colon at day 67. The DNA damage marker p-H2AX is shown. Scale bar: 50 μm. (**B**) Statistical analysis of p-H2AX positive cells in each field (×400). Data represent mean ± SEM (n = 4). ***p < 0.001. (**C**) Western blotting analysis of whole distal colon at day 67. The original blots are presented in Supplementary Fig. S7. AOM, azoxymethane; DSS, dextran sodium sulfate; N, neomycin; SEM, standard error of the mean; V, vancomycin; V/N, vancomycin and neomycin.

To assess the effect of antibiotics on colitis and colon carcinogenesis, we employed a CAC model. The carcinogen AOM was injected in C57BL/6 mice followed by three rounds of 2% dextran sodium sulfate (DSS) in drinking water. Throughout the AOM/DSS treatment, mice were orally administrated a combination of vancomycin and neomycin as broad-spectrum antibiotics or either vancomycin or neomycin alone. Tumor formation was analyzed at day 67 of AOM/DSS treatment (Fig. 1A). In the absence of antibiotic treatment, AOM/DSS-treated mice exhibited a high tumor burden in the colon-rectum regions at day 67 (Fig. 1B,C). While neomycin treatment alone did not affect tumor formation, vancomycin with or without neomycin markedly reduced AOM/DSS-induced CAC development (Fig. 1B,C). To analyze the pathology of AOM/DSS-induced tumors, we performed hematoxylin and eosin (H&E) staining of colon tissue at day 67 of AOM/DSS treatment. Histological analysis showed that vancomycin/neomycin or vancomycin alone greatly suppressed the development of adenocarcinoma induced by AOM/DSS treatment (Fig. 1D). Immunohistochemical evaluation confirmed that the activation of β-catenin and c-myc in the AOM/DSS-treated colon epithelia was reduced by vancomycin/neomycin or vancomycin alone (Supplementary Fig. S1A). Vancomycin treatment also decreased the epithelial proliferation in AOM/DSS-treated mice as shown by immunohistochemical analysis of cyclin D1 and Ki-67 (Supplementary Fig. S1A,B). Moreover, we found that the expression of the phosphorylated histone p-H2AX, a marker for double-stranded DNA breaks, was increased in AOM/DSS-treated control colons and neomycin-treated colons, and its expression was greatly reduced by vancomycin/neomycin or vancomycin treatment (Fig. 2A,B). The expression of inducible nitric oxide synthase (iNOS), apoptosis-related protein Bax, Bcl2, and phosphorylated p53 was induced by AOM/DSS treatment, and their expression was suppressed by vancomycin but not neomycin (Fig. 2C). These results indicate that vancomycin inhibits DNA damage, apoptosis, and proliferation of epithelial cells, and development of CAC.

### Vancomycin reduced the infiltration of neutrophils to colon tumors

**Figure 3:**
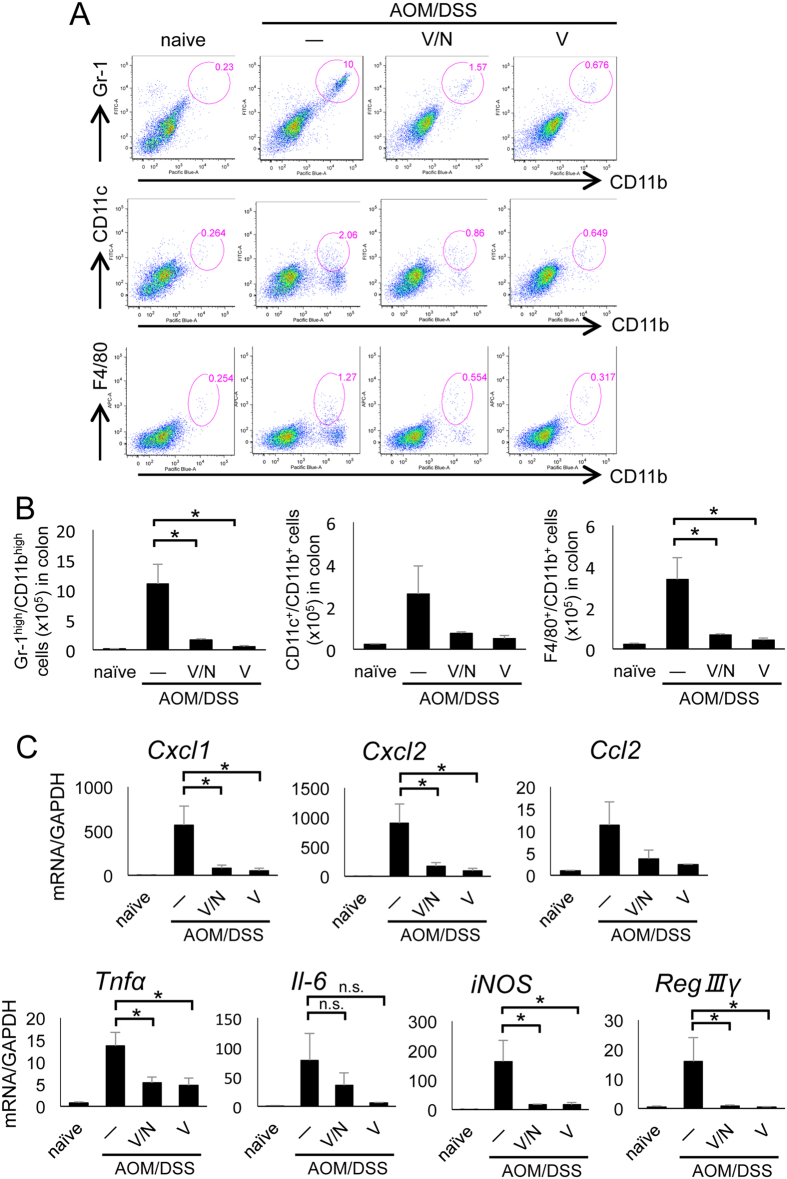
Vancomycin suppressed infiltration of neutrophil to colon tumors. (**A**) Flow cytometric analysis of immune cells in the lamina propria of the colon at day 67. Single cell suspensions were stained with FITC-conjugated anti-Gr-1, eFluor450-conjugated anti-CD11b, FITC-conjugated anti-CD11c, or APC-conjugated anti-F4/80 and analyzed on a FACS Canto flow cytometer as described in the Materials and Methods. (**B**) Statistical analysis of flow cytometry. (**C**) Real-time PCR analysis of distal colon at day 67. AOM, azoxymethane; APC, allophycocyanin; DSS, dextran sodium sulfate; FITC, fluorescein isothiocyanate; n.s., not significant; SEM, standard error of the mean; V, vancomycin; V/N, vancomycin and neomycin. Data represent mean ± SEM (n = 4). *p < 0.05.

To further delineate the effect of vancomycin treatment on CAC development, we evaluated the migration ability of myeloid cells such as neutrophils (Gr-1^high^/CD11b^high^), dendritic cells (CD11c^+^/CD11b^+^), and macrophages (F4/80^+^/CD11b^+^) to colon cancer induced by AOM/DSS treatment with or without vancomycin. At day 67 after AOM/DSS treatment, we observed that a large number of neutrophils were recruited to the AOM/DSS-treated colon. Vancomycin treatment greatly reduced the infiltration of neutrophils, although we observed a small remaining number of macrophages and dendritic cells (Fig. 3A,B). In bone marrow, although CD11c^+^/CD11b^+^ dendritic cells and F4/80^+^/CD11b^+^ macrophages were not changed by AOM/DSS treatment, Gr-1^high^/CD11b^high^ neutrophils were enhanced at day 67 of AOM/DSS treatment, and vancomycin treatment reduced the expansion of these neutrophils (Supplementary Fig. S2). To understand whether the number of neutrophils was increased by tumors or by colitis, we analyzed the number of neutrophils (Gr-1^high^/CD11b^high^) during the course of AOM/DSS-induced carcinogenesis in colon, bone marrow, and spleen by flow cytometric analysis. From the onset of treatment, AOM/DSS markedly increased the number of neutrophils not only in colon but also in bone marrow and spleen (Supplementary Fig. S3). These results suggest that AOM/DSS treatment induces long-term infiltration of neutrophils into the colon, which might prolong inflammation and promote colon carcinogenesis. Furthermore, bone marrow and spleen appear to continuously prepare neutrophil precursors and contribute to colon inflammation. These data provide the evidence that vancomycin reduces the neutrophil migration from bone marrow and spleen to the inflamed colon in the development of CAC.

### Vancomycin treatment attenuated colon inflammation

We next measured the expression of mRNA for chemokines and pro-inflammatory mediators in the colon using real-time PCR analysis. AOM/DSS-treated mice showed elevated expression of the chemokines CXCL1, CXCL2, and CCL2, and vancomycin treatment inhibited the expression of these chemokines (Fig. 3C). Moreover, we observed higher mRNA expression of the pro-inflammatory mediators tumor necrosis factor α (TNFα), interleukin-6 (IL-6), and iNOS in AOM/DSS-treated colons. The mRNA levels of TNFα and iNOS were significantly downregulated by vancomycin treatment (Fig. 3C). The expression of IL-6 appeared to be downregulated by vancomycin treatment but was not significant (Fig. 3C). The expression of regenerating islet-derived protein 3 gamma (RegIIIγ), which is related to tissue damage and repair, was also strongly induced in AOM/DSS-treated colons, and vancomycin treatment significantly reduced its expression (Fig. 3C). Taken together, these data suggest that vancomycin-sensitive bacteria caused recruitment of neutrophils, which promoted TNFα and iNOS expression, followed by epithelial tissue damage, DNA damage, and tumor growth.

### Vancomycin could decrease the severity of colitis

**Figure 4:**
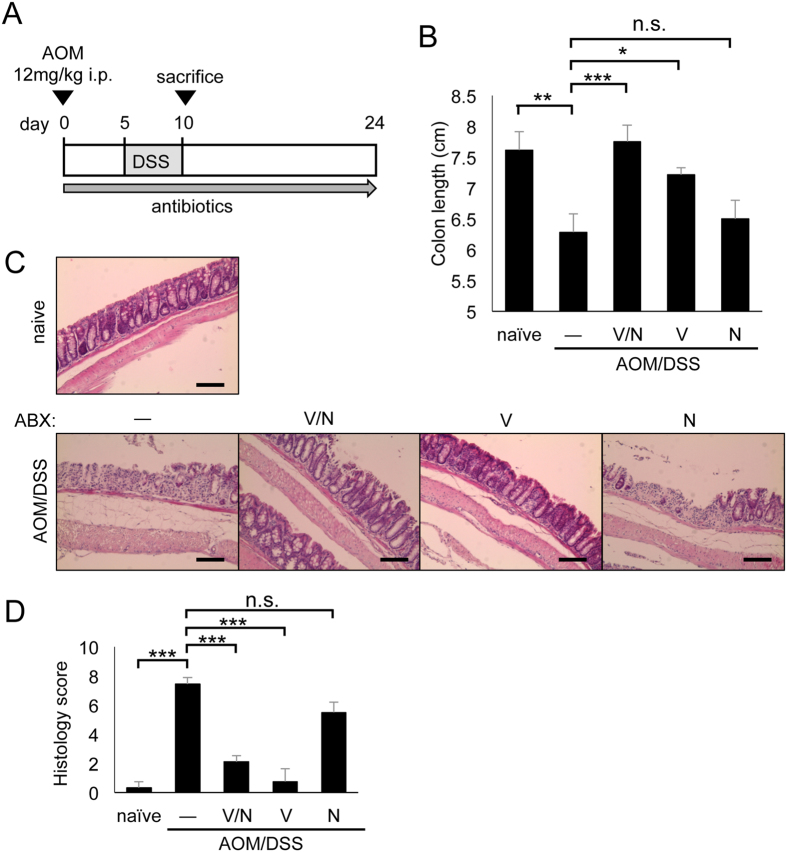
Vancomycin could decrease the severity of colitis. (**A**) Schematic of administration of antibiotics to AOM/DSS-treated mice. (**B**) Statistical analysis of colon length at day 10 (n = 6). (**C**) H&E staining of distal colon at day 10. Scale bar: 100 μm. D. Histology scores of colons at day 10 (n = 6). ABX, antibiotics; AOM, azoxymethane; DSS, dextran sodium sulfate; H&E, hematoxylin and eosin; i.p., intraperitoneal; N, neomycin; n.s., not significant; SEM, standard error of the mean; V, vancomycin; V/N, vancomycin and neomycin. Data represent mean ± SEM. *p < 0.05, **p < 0.01, ***p < 0.001.

Because we observed that neutrophil infiltration and inflammation were induced in the colon at day 67 after AOM/DSS treatment, which was reduced by vancomycin, next we examined the contribution of vancomycin-sensitive bacteria in the colon early in the time course of AOM/DSS treatment (Fig. 4A). At day 10, a shorter colon length was found in AOM/DSS-treated mice (Fig. 4B). Administration of vancomycin/neomycin or vancomycin alone could inhibit the colon shortage induced by AOM/DSS, whereas treatment with neomycin alone did not (Fig. 4B). Histological analysis also revealed that the combination of vancomycin/neomycin or vancomycin alone reduced colon inflammation, such as ulceration and infiltration, but that neomycin did not affect the severity of colitis (Fig. 4C,D). These results further support the idea that vancomycin-sensitive bacteria play a critical role in the severity of colitis.

**Figure 5:**
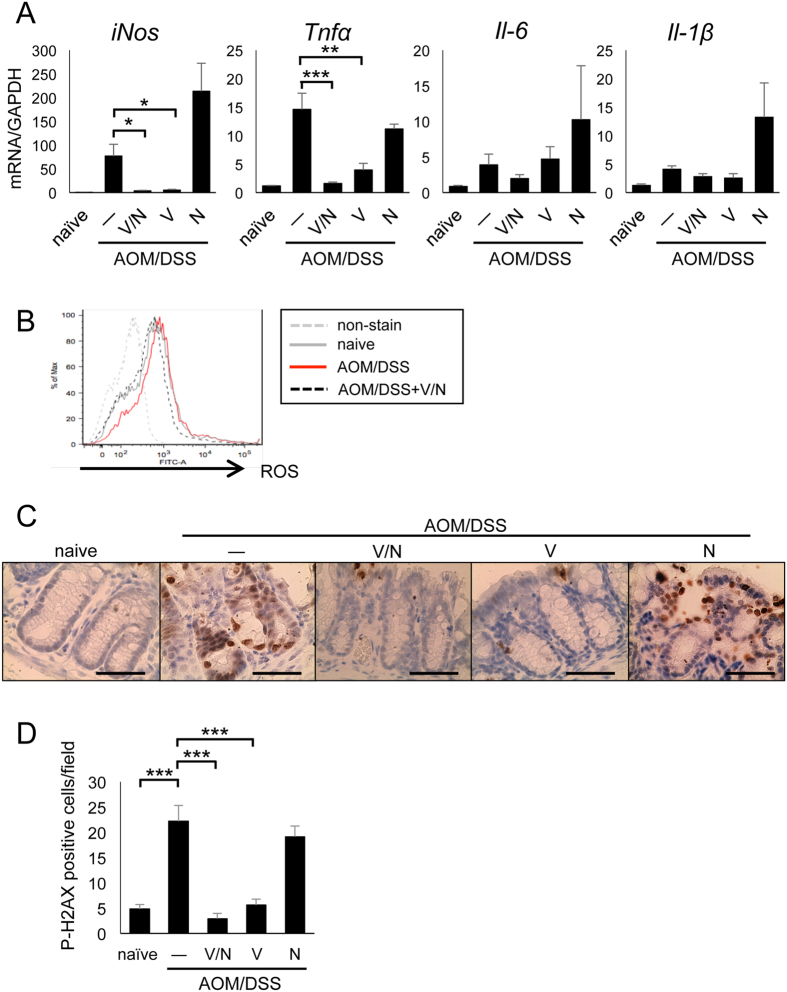
Vancomycin reduced DNA damage in colon epithelial cells through downregulating pro-inflammatory mediator expression. (**A**) Real-time PCR analysis of distal colon at day 10. (**B**) ROS production from lamina propria-infiltrated cells at day 10. (**C**) Immunohistochemical analysis of distal colon at day 10. The DNA damage marker p-H2AX is shown. Scale bar: 50 μm. (**D**) Statistical analysis of p-H2AX positive cells in each field (×400). AOM, azoxymethane; DSS, dextran sodium sulfate; N, neomycin; ROS, reactive oxygen species; SEM, standard error of the mean; V, vancomycin; V/N, vancomycin and neomycin. Data represent mean ± SEM (n = 6). *p < 0.05, **p < 0.01, ***p < 0.001.

We also examined the expression of pro-inflammatory mediators at day 10 (inflammatory phase) of AOM/DSS treatment. Vancomycin/neomycin or vancomycin alone greatly downregulated iNOS and TNFα mRNA expression, but not that of IL-6 and IL-1β at day 10 (Fig. 5A). Because iNOS catalyzes the production of toxic reactive nitrogen species and elevates DNA damage and apoptosis, we examined the production of reactive oxygen species (ROS) in the AOM/DSS-treated colons. ROS production was reduced by vancomycin/neomycin treatment (Fig. 5B). Moreover, the DNA damage marker p-H2AX was highly induced at day 10 of AOM/DSS treatment and was decreased by treatment with vancomycin/neomycin or vancomycin alone (Fig. 5C,D). These results suggest that vancomycin-sensitive bacteria induce iNOS and TNFα production in damaged colon tissue, which leads to DNA damage and apoptosis in colon epithelial cells.

### Vancomycin inhibited the recruitment of neutrophils into AOM/DSS-treated colon tissue

**Figure 6:**
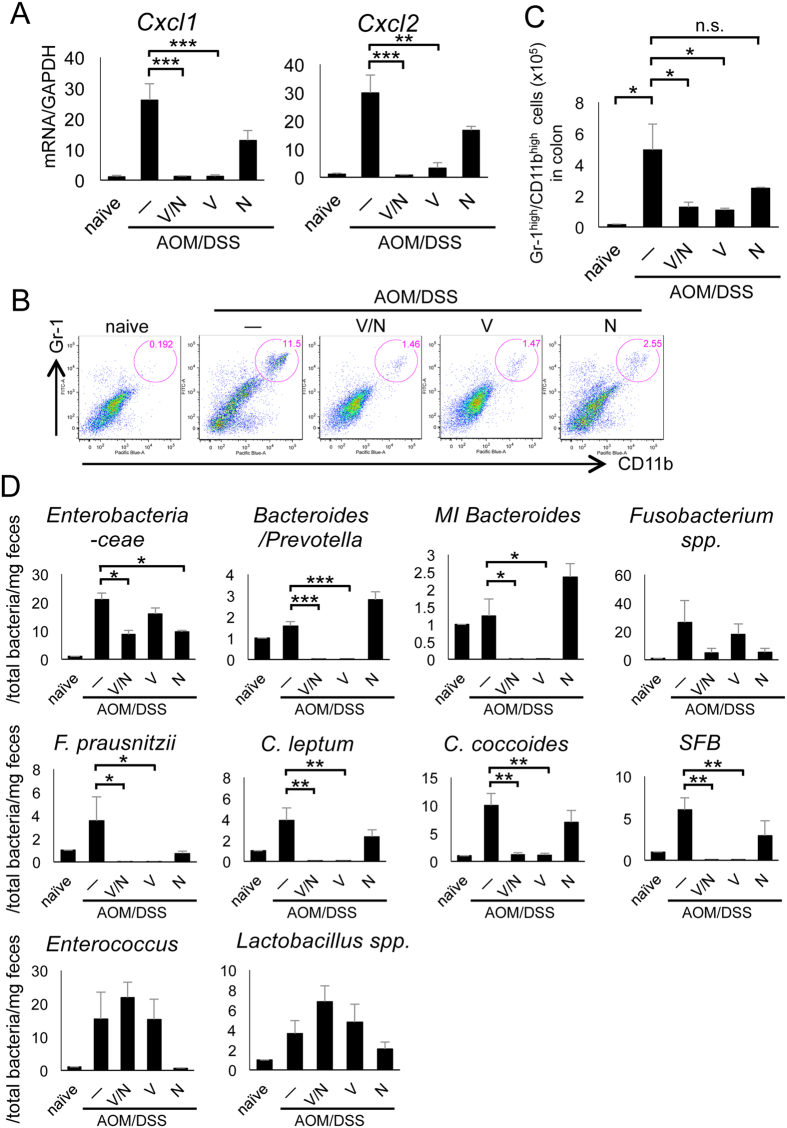
Vancomycin inhibited the recruitment of myeloid cells into inflamed colon tissue. (**A**) Real-time PCR analysis for chemokines in the distal colon at day 10. (**B**) Flow cytometric analysis of immune cells into the lamina propria of the colon at day 10. Single cell suspensions were stained with FITC-conjugated anti-Gr-1 and eFluor450-conjugated anti-CD11b and analyzed with a FACS Canto flow cytometer as described in the Materials and Methods. (**C**) Statistical analysis of flow cytometry. (**D**) Relative presence of bacteria in feces was determined by real-time PCR. DNA samples were extracted from feces at day 24 of AOM/DSS treatment, and the amount of total bacteria was determined per unit weight (total bacteria/mg feces). Quantities of each bacterium were normalized to the amount of total bacteria in each sample (n = 4–6). AOM, azoxymethane; DSS, dextran sodium sulfate; FITC, fluorescein isothiocyanate; N, neomycin; n.s., not significant; SEM, standard error of the mean; V, vancomycin; V/N, vancomycin and neomycin. Data represent mean ± SEM (n = 5). *p < 0.05, **p < 0.01, ***p < 0.001.

As we had observed neutrophil infiltration to the colon at day 67 of AOM/DSS treatment, we evaluated neutrophil infiltration to AOM/DSS-treated colons at the earlier time point (day 10). AOM/DSS-treated mice showed an elevated expression of the chemokines CXCL1 and CXCL2, which induce neutrophil recruitment, whereas vancomycin treatment inhibited the expression of these chemokines (Fig. 6A). Indeed, AOM/DSS treatment strongly induced the infiltration of neutrophils at day 10 of AOM/DSS treatment (Fig. 6B,C). Vancomycin administration to the AOM/DSS-treated mice greatly reduced the infiltration of neutrophils to the colon at day 10, but neomycin treatment did not (Fig. 6B,C). AOM/DSS-induced tissue injury and colon infiltration with neutrophils were sustained until day 24 (Supplementary Fig. S4A–C). In bone marrow and spleen, Gr-1^high^/CD11b^high^ neutrophils at day 24 were enhanced by AOM/DSS treatment (Supplementary Fig. S5A,B). We confirmed that the Gr-1^high^/CD11b^high^ cells that were induced by AOM/DSS treatment were neutrophils with ring-shaped nuclei (Supplementary Fig. S6A). They produced ROS, iNOS, and several pro-inflammatory cytokines in colitis (Supplementary Fig. S6B,C).

Because of the observed effects of the antibiotic vancomycin on AOM/DSS-treated colon tissues, we examined the bacterial composition of the colon at day 24 of AOM/DSS treatment using real-time PCR to analyze bacterial 16S ribosomal DNA. AOM/DSS-treated mice exhibited a marked increase in the amount of Gram-positive bacteria, including *Clostridium leptum, C. coccoides*, and *segmented filamentous bacteria* (*SFB*), as well as Gram-negative *Enterobacteriaceae* in their stool (Fig. 6D). We confirmed that vancomycin treatment dramatically reduced the amount of Gram-positive bacteria, including *Faecalibacterium prausnitzii, C. leptum, C. coccoides* and *SFB*. In addition, vancomycin treatment reduced the number of the anaerobic Gram-negative bacteria *Bacteroides/Prevotella* and *mouse intestinal Bacteroides* (*MI Bacteroides*), while neomycin treatment did not. On the other hand, the number of aerobic Gram-negative *Enterobacteriaceae* was significantly reduced by treatment with neomycin but not vancomycin (Fig. 6D). In addition, levels of Gram-positive *Enterococcus* and *Lactobacillus spp.* and anaerobic Gram-negative *Fusobacterium spp.* were slightly increased by AOM/DSS treatment, but they were not significantly reduced by vancomycin or neomycin (Fig. 6D). These results suggest that vancomycin-sensitive bacteria, such as *C. leptum, C. coccoides, SFB*, and *Bacteroides*, in the colon might trigger the migration of neutrophils to the inflamed colon from bone marrow and spleen. The neutrophil-produced iNOS then causes epithelial DNA damage, which in turn promotes colon carcinogenesis.

### Aminoguanidine (AG) treatment inhibited the initiation of CAC

**Figure 7:**
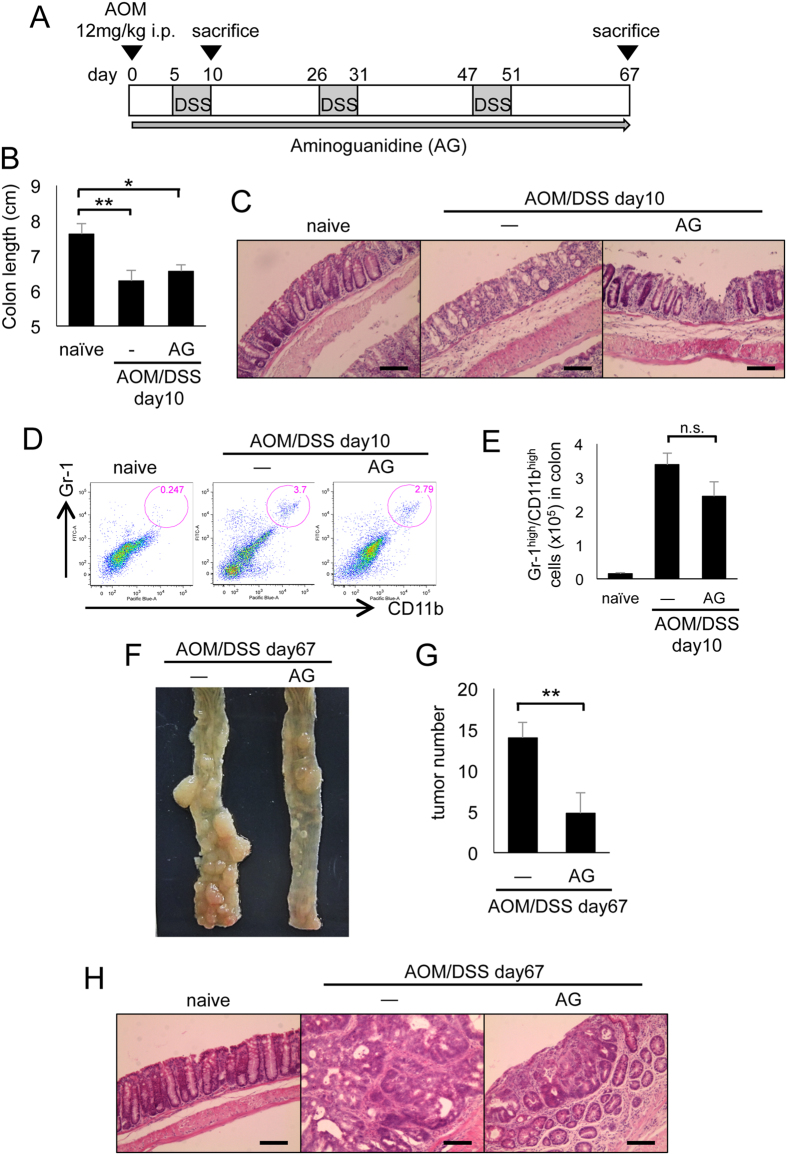
Aminoguanidine treatment inhibits the initiation of colitis associated colon cancer. (**A**) Schematic of mice treated with AOM/DSS. After initial AOM injection (12 mg/kg), DSS was given in drinking water followed by regular water three times. AG was administered throughout this procedure. (**B**) Statistical analysis of colon length at day 10. (**C**) H&E staining of distal colon at day 10. Scale bar: 100 μm. (**D**) Flow cytometric analysis of immune cells in the lamina propria of the colon at day 10. Single cell suspensions were stained with FITC-conjugated anti-Gr-1 and eFluor450-condugated anti-CD11b, and were analyzed on a FACS Canto flow cytometer as described in Materials and Methods. (**E**) Statistical analysis of flow cytometry (n = 3–5). (**F**) Macroscopic view of tumor formation in colon-rectum regions of AOM/DSS-treated control mice and vancomycin-treated mice at day 67. (**G**) Statistical analysis of tumor number in colon-rectum regions at day 67. (**H**) H&E staining of distal colon at day 67. Scale bar: 100 μm. AG, aminoguanidine; AOM, azoxymethane; DSS, dextran sodium sulfate; FITC, fluorescein isothiocyanate; H&E, hematoxylin and eosin; i.p., intraperitoneal; N, neomycin; n.s., not significant; SEM, standard error of the mean; V, vancomycin; V/N, vancomycin and neomycin. Data represent mean ± SEM (n = 5). *p < 0.05, **p < 0.01.

To investigate the effect of iNOS on colitis and colitis-associated carcinogenesis, we treated mice with the iNOS inhibitor AG upon injection of AOM (12 mg/kg), followed by three rounds of DSS treatment (Fig. 7A). At day 10, AG-treated mice exhibited colon shortness similar to the AOM/DSS-treated control mice (Fig. 7B). Furthermore, H&E staining of the colon at day 10 revealed that AG-treated mice also showed severe colon tissue damage (Fig. 7C). By flow cytometric analysis, we also found that the infiltration of neutrophils in AOM/DSS-treated colons was similar regardless of AG treatment (Fig. 7D,E). However, at the end of AOM/DSS treatment (at day 67), AOM/DSS/AG-treated mice had fewer tumors compared with AOM/DSS-treated control mice (Fig. 7F–H). These results indicate that iNOS has no effect on the severity of colitis but promotes AOM/DSS-induced carcinogenesis.

Taken together, vancomycin treatment suppressed the recruitment of Gr-1^high^/CD11b^high^ neutrophils caused by AOM/DSS treatment, and that reduced the severity of colitis and prevented CAC development. Hence, we suggest that vancomycin-sensitive bacteria trigger colitis and colon cancer in the murine CAC model.

## Discussion

Here we showed that vancomycin dramatically suppressed inflammation-mediated tumorigenesis in the AOM/DSS-induced murine CAC model. We confirmed that vancomycin dramatically reduced the number of Gram-positive bacteria, such as *F. prausnitzii, C. leptum, C. coccoides* and *SFB*, as well as the anaerobic Gram-negative bacteria *Bacteroides/Prevotella* and *MI Bacteroides*, while neomycin did not. Thus, these results suggest that vancomycin-sensitive bacteria trigger colon inflammation and initiation of CAC. Our results may contrast with previous reports showing that Gram-negative bacteria *Enterobacteriaceae*, including *Escherichia coli*, induced inflammatory bowel disease such as Crohn’s disease^26^,^27^,^28^,^29^,^30^,^31^. In our report, vancomycin treatment did not reduce the number of *Enterobacteriaceae* but dramatically reduced colon inflammation and carcinogenesis; neomycin, which kills *Enterobacteriaceae*, did not affect the severity of colitis or tumor multiplicity.

Our results showed that commensal bacteria induced the production of the chemokines CXCL1 and CXCL2, which induced the infiltration of Gr-1^high^/CD11b^high^ neutrophils in the AOM/DSS-induced colitis. In addition, we showed that during the course of AOM/DSS treatment, the number of Gr-1^high^/CD11b^high^ neutrophils increased greatly in bone marrow and spleen and appear to migrate to the injured colon. High expression of TNFα, iNOS, and ROS indicated that these neutrophils were pro-inflammatory. Previously, we demonstrated that Gr-1^high^/CD11b^high^ cells in the colon, bone marrow, and spleen were induced in an DSS-induced murine colitis model^22^,^23^. We have shown that infusion of neutrophils early in DSS-induced colitis reduces inflammation-induced tissue damage^22^, and the absence of neutrophils early in DSS-induced colitis worsens tissue damage^23^. Our previous reports have shown that Gr-1^high^/CD11b^high^ cells engulf translocated bacteria early in DSS-induced colitis, which contributes to early recovery from colitis^22^,^23^. In contrast to this complete recovery from colitis from one round of treatment with DSS, here with repeated AOM/DSS treatment we observed damage to colon tissue by prolonged infiltration of Gr-1^high^/CD11b^high^ neutrophils against the invasion of bacteria. Others have also reported that the absence of neutrophils reduced tumor multiplicity in the AOM/DSS-induced murine CAC model^32^,^33^. In these reports, Ly6G^+^/CD11b^+^ cells (Gr-1^high^/CD11b^high^ cells) were recognized as myeloid-derived suppressor cells (MDSCs). Generally MDSCs have an M2-like phenotype, producing IL-10 and arginase. However, our neutrophils are pro-inflammatory cells that produce ROS, iNOS, and several pro-inflammatory cytokines in colitis (Supplementary Fig. S6B,C). In this study, we showed that vancomycin treatment inhibited infiltration of neutrophils to the inflamed colon and suppressed colitis and colon carcinogenesis. Our results suggest that vancomycin-sensitive bacteria attract pro-inflammatory Gr-1^high^/CD11b^high^ neutrophils, which causes colitis and colon cancer.

The results here show that vancomycin treatment reduced TNFα and iNOS production in the colon. Recent studies using mouse models of inflammation-associated cancer, including CAC, have shown that inflammation mainly acts as tumor promoter^24^,^34^,^35^. Inflammatory mediators such as TNFα promote intestinal epithelial proliferation during CAC induction^24^. Erdman *et al*. reported that the presence of Gr-1^+^ neutrophils and elevated nitric oxide (NO) and TNFα trigger colonic inflammation and carcinogenesis in Rag2-deficient mice^36^. In our experiments, we demonstrated that vancomycin-sensitive bacteria induced infiltration of neutrophils, which produced iNOS and TNFα, and promoted inflammation-mediated tumorigenesis in the AOM/DSS-induced murine CAC model. Because this murine model is known to be similar to human ulcerative colitis, our results are valuable for understanding the mechanism of initiation of human ulcerative colitis and colon cancer.

In the murine model of colorectal cancer induced by loss of function of adenomatous polyposis coli (APC), it was reported that inhibition of two major enzymes, cyclooxygenase-2 (Cox2) and iNOS, suppressed tumor formation^37^,^38^,^39^. Also, experimental colitis was shown to increase tumorigenesis in APC+/Min mice through an iNOS-dependent mechanism^40^. iNOS is a primary regulator of NO production in innate immune cells, and NO contributes to bactericidal activities. NO reacts with superoxide radicals, resulting in formation of peroxynitrite, which generates nitrate^41^. Peroxynitrite not only kills bacteria, but also induces direct DNA damage in host intestinal epithelial cells, and enhances tumor initiation^39^. In our results, vancomycin treatment strongly reduced iNOS production and inhibited recruitment of iNOS-producing neutrophils induced by AOM/DSS treatment. Furthermore, colitis-induced colon tissue damage and DNA damage were dramatically suppressed by vancomycin treatment. We also confirmed the effect of iNOS in colitis and CAC development using the iNOS inhibitor AG. Previously, whether iNOS improves or exacerbates colitis was unclear^42^. Here, we clearly showed that iNOS did not affect the severity of colitis but played a role in initiation of colon cancer by contributing to DNA damage.

In conclusion, our results suggest that vancomycin-sensitive bacteria induced colon inflammation and DNA damage by attracting neutrophils to the damaged colon tissue, and finally, promoted tumor formation.

## Materials and Methods

### Mice

All experiments used C57BL/6 NCrSlc female mice, 6–8 weeks old, which were purchased from SLC (Japan SLC Inc., Shizuoka, Japan) and maintained at the Animal Research Facility at Nagoya University Graduate School of Medicine under specific pathogen-free conditions. This work was approved by the ethical committee of Nagoya University. All experiments were performed in accordance with the approved guidelines.

### Induction of colorectal cancer

Mice were intraperitoneally injected with 12 mg/kg body weight of AOM (Sigma-Aldrich, St Louis, MO, USA) dissolved in phosphate-buffered saline (PBS). Five days after AOM administration, 2% DSS (MP Biomedicals, Santa Ana, CA, USA, molecular weight 35,000–50,000 kDa) was administered through the drinking water for 5 consecutive days (first round of DSS treatment). Subsequently, untreated water was given for 16 days. Then DSS was administered for 5 days followed by 16 days of untreated water (second round of DSS treatment). Finally, DSS was administered for 4 days (third round of DSS treatment) (Fig. 1A). Body weight was assessed at least 3 days per week throughout the course of the experiment.

### Antibiotic and AG administration

Mice were treated with neomycin (1 g/L; Wako, Osaka, Japan), vancomycin (500 mg/L; Wako), or AG (2 g/L; Sigma-Aldrich) in drinking water.

### Histopathological analysis

Mouse colon tissues were prepared as Swiss rolls fixed in 4% paraformaldehyde neutral buffer solution for paraffin embedding. Paraffin-embedded tissues were cut into 4 μm sections and stained with H&E. Histology scores were determined as described in a previous report^14^ and were assigned based on the extent and severity of inflammation, ulceration, and hyperplasia of the mucosa in the distal colon.

### Lamina propria cell isolation

Colons were opened longitudinally, cut into fine pieces, and incubated with Hanks’ balanced salt solution (HBSS) containing 5 mM ethylenediamine tetraacetic acid for 15 min at 37 °C to remove epithelial cells. Colonic pieces without epithelial cells were then incubated with PBS containing 4% fetal bovine serum (FBS), 0.5 mg/mL collagenase type II, 1 mg/mL dispase, and 50 μg/mL DNase for 20 min at 37 °C. The cells were washed twice and suspended in PBS.

### Flow cytometric analyses and cell sorting

The cells were washed twice and cells (1 × 10^6^) were suspended in 50 μL PBS supplemented with 1% FBS and stained for 20 min at 4 °C with directly conjugated fluorescent antibodies (1:500). Antibodies were as follows: eFluor450-conjugated anti-CD11b (eBioscience, Santa Clara, CA, USA), fluorescein isothiocyanate (FITC)-conjugated anti-Gr-1 (BD Biosciences, Franklin Lakes, NJ, USA), allophycocyanin (APC)-conjugated anti-Gr-1 (eBioscience), FITC-conjugated anti-CD11c (eBiosciences), APC-conjugated anti-F4/80 (eBioscience), isotype control Rat IgG2a, and isotype control Rat IgG2b (BD Biosciences). Stained cells were analyzed with a FACS Canto flow cytometer using FACS Diva software (BD Biosciences), and the data were analyzed with FlowJo software (TreeStar, Ashland, OR, USA). Cells were sorted with a FACS Aria flow cytometer (BD Biosciences), washed twice, and suspended in PBS. Cells (2 × 10^5^) were resuspended in 100 μL PBS and centrifuged onto microscopic slides using a Cytospin-4 (Thermo Scientific, Waltham, MA, USA). Slides were then stained by May-Grunwald Giemsa according to a standard protocol.

### RT-PCR analysis

Total RNA was isolated using the RNeasy mini kit (Qiagen, Hilden, Germany) according to the manufacturer’s recommended protocol. Total RNA from a small number of cells was isolated using RNAqueous^®^-Micro Kit (Ambion, Austin, TX, USA). Residual genomic DNA was digested and removed using DNaseI (Invitrogen, Waltham, MA, USA). First-strand cDNA was synthesized using 1 μg total RNA and a High Capacity cDNA Reverse Transcription Kit (Applied BioSystems, Waltham, MA, USA) for RT-PCR. RT-PCR was performed using the Takara EX Taq (Takara, Shiga, Japan) according to the manufacturer’s instructions. Real-time RT-PCR was performed using SYBR green (TOYOBO, Osaka, Japan) according to the manufacturer’s instructions. Expression data were normalized to GAPDH mRNA expression. The primer sequences are shown in Supplementary Table 1.

### Western blot analysis

Tissue samples were homogenized in standard radioimmunoprecipitation assay buffer with phenylmethylsulfonyl fluoride. Protein lysates were separated by 6–10% SDS-gel electrophoresis, and transferred to Immobilon-P membranes (Millipore, Darmstadt, Germany). Membranes were blocked in PBS with Tween buffer containing 3% skim milk for 1 hour at room temperature, probed with primary antibodies and secondary horseradish peroxidase-conjugated antibodies (GE Healthcare, Little Chalfont, UK), and developed using an enhanced chemiluminescence Western blot detection reagent (GE Healthcare).

### 16S rRNA gene-quantitative PCR analysis

16S rRNA gene-quantitative PCR was performed as previously described^43^. Bacterial genomic DNA was isolated from fecal pellets using the QIAamp DNA Stool Mini Kit (Qiagen). Real-time PCR was performed using SYBR green (TOYOBO) according to the manufacturer’s instructions. Data were normalized to those of total bacteria. The primer sequences are shown in Supplementary Table 1.

### ROS production analysis

Flow cytometry was used to measure intracellular ROS accumulation with 5-(and 6-) chloromethyl–2′, 7′-dichlorodihydrofluorescein diacetate, acetyl ester (CM-H2DCFDA) (Invitrogen). Isolated lamina propria cells were incubated in RPMI medium (Sigma-Aldrich) containing 5 μM CM-H2DCFDA for 30 min at 37 °C in the dark. Fluorescence was analyzed with a FACS Canto flow cytometer using FACS Diva software (BD Biosciences), and the data were analyzed with FlowJo software (TreeStar).

### Statistical analysis

Data are expressed as mean ± standard error of the mean (SEM). The statistical analysis was carried out by one-way analysis of variance (ANOVA) followed by post-hoc analysis. P values less than 0.05 were considered statistically significant.

## Additional Information

**How to cite this article**: Tanaka, Y. *et al*. Vancomycin-sensitive bacteria trigger development of colitis-associated colon cancer by attracting neutrophils. *Sci. Rep.* **6**, 23920; doi: 10.1038/srep23920 (2016).

## Supplementary Material

Supplementary Information
