# Supplementary material for: Vancomycin-sensitive bacteria trigger development of colitis-associated colon cancer by attracting neutrophils
Source: Sci Rep. 2016 Apr 6;6:23920. doi: 10.1038/srep23920 (PMC4822119; doi:10.1038/srep23920)
Supplement: Supplementary Information [file srep23920-s1.pdf]

Supplementary information

Vancomycin-sensitive bacteria trigger development of colitis-associated colon cancer by attracting neutrophils

Yuriko Tanaka[1], Sachiko Ito[1] and Ken-ichi Isobe[1, 2] *

[1]Department of Immunology, Nagoya University Graduate School of Medicine, 65 Tsurumai-cho, Showa-ku, Nagoya, Aichi, 466-8550, Japan
[2]Department of Food Science and Nutrition, Nagoya Women's University 3-40 Shioji-cho, Mizuho-ku, Nagoya, Aichi, 467- 8610, Japan
*Address correspondence and reprint requests to Dr. K. Isobe, Department of Food Science and Nutrition, Nagoya Women's University 3-40 Shioji-cho, Mizuho-ku, Nagoya, Aichi, 467- 8610, Japan.
Email address: isobe@nagoya-wu.ac.jp
Tel: +81-52-852-9425
Fax: +81-52-852-7470

Supplementary Table 1. The following primers were used for RT-PCR.

genes	sequences (Forward ; 5'-3')	sequences (Reverse ; 5'-3')
Tnfa	GCCCATATACCTGGGAGGAG	CACCCATTCCCTTCACAGAG
Il-6	CCGGAGAGGAGACTTCACAG	TCCACGATTTCCCAGAGAAC
iNOS	AGGGAATCTTGGAGCGAGTTG	AGTAGCTGCCGCTCTCATC
RegⅢγ	CCTGATGCTCCTTTCTCAGG	ATGTCCTGAGGGCCTCTTTT
Gapdh	AACTTTGGCATTGTGGAAGG	ACACATTGGGGGTAGGAACA
Il-1β	GCCCATCCTCTGTGACTCAT	AAGGCCACAGGTATTTTGTCG
Cxcl1	GCTGGGATTCACCTCAAGAA	TCTCCGTTACTTGGGGACAC
Cxcl2	TCCAGAGCTTGAGTGTGACG	AGGCACATCAGGTACGATCC
Ccl2	TGAATGTGAAGTTGACCCGT	AAGGCATCACAGTCCGAGTC
Total bacteria	GGTGAATACGTTCCCGG	TACGGCTACCTTGTTACGACTT
Enterobacteriaceae	TGCCGTAACTTCGGGAGAAGGCA	TCAAGGACCAGTGTTCAGTGTC
Bacteroides/Prevotella	CCTWCGATGGATAGGGGTT	CACGCTACTTGGCTGGTTCAG
MI Bacteroides	CCAGCAGCCGCGGTAATA	CGCATTCCGCATACTTCTC
Fusobacterium spp.	CWAACGCGATAAGTAATC	TGGTAACATACGAAAGGG
Faecalibacterium prausnitzii	CCCTTCAGTGCCGCAGT	GTCGCAGGATGTCAAGAC
Clostridium leptum	CCTTCCGTGCCGSAGTTA	GAATTAAACCACATACTCCACTGCTT
Clostridium coccoides	AAATGACGGTACCTGACTAA	CTTTGAGTTTCATTCTTGCGAA
SFB	GACGCTGAGGCATGAGAGCAT	GACGGCACGGATTGTTATTCA
Enterococcus	CCTTATTGTTAGTTGCCATCATT	ACTCGTTGTACTTCCCATTGT
Lactobacillus spp.	AGCAGTAGGGAATCTTCCA	CACCGCTACACATGGAG

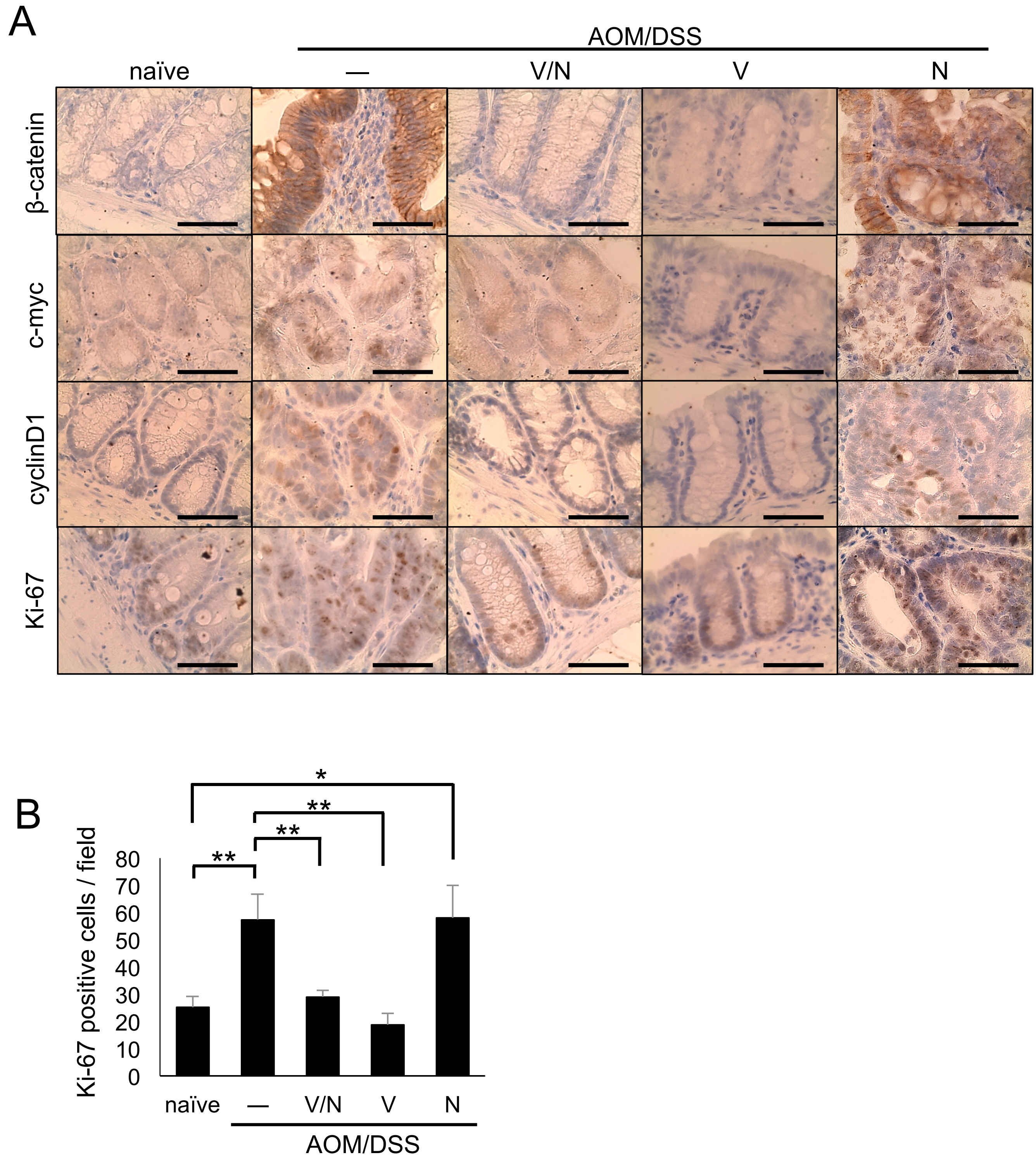

Supplementary figure S1. Immunohistochemical analysis of CAC

A. Immnohistochemical analysis of distal colon at day 67. β-catenin, NFκB p65, c-myc, cyclinD1, Ki-67 were shown. Scale bar: 50 μm.

B. Statistical analysis of Ki-67 positive cells in each field (x400). Data represent mean ± SEM (n = 4). $^{**}p < 0.01$.

V/N, vancomycin and neomycin; V, vancomycin; N, neomycin.

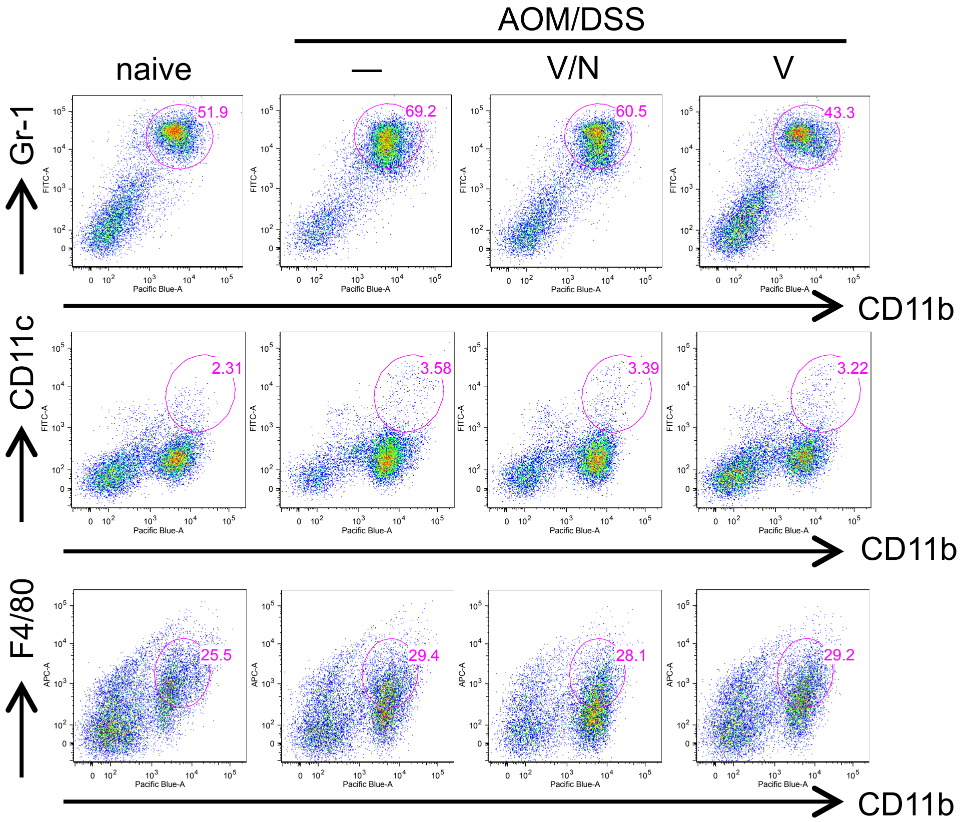

Supplementary figure S2. Vancomycin suppressed the increase of Gr-1^{high}/CD11b^{high} neutrophils in bone marrow of AOM/DSS-treated mice

Bone marrow cells were removed from AOM/DSS treated mice at day 67. Single cell suspensions of these tissues were stained with FITC-conjugated anti-Gr-1, eFluor450-conjugated anti-CD11b, FITC-conjugated anti-CD11c, APC-conjugated anti-F4/80, and analyzed on a FACS Canto flow cytometer as described in the Materials and Methods. V/N, vancomycin and neomycin; V, vancomycin.

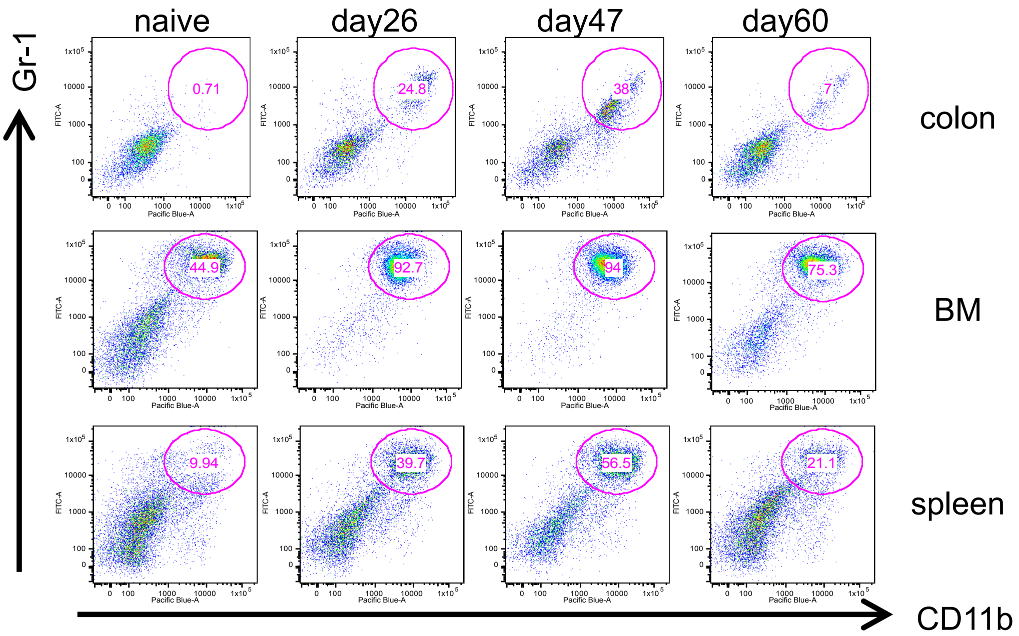

Supplementary figure S3. AOM/DSS treatment induced migration of myeloid cells
Three rounds of DSS treatment following AOM injection. Flow cytometric analysis of immune cells in lamina propria of colon, bone marrow cells and splenic cells at the indicated time point. Single cell suspensions were stained with FITC-conjugated anti-Gr-1, eFluor450-conjugated anti-CD11b and analyzed on a FACS Canto flow cytometer as described in the Materials and Methods.

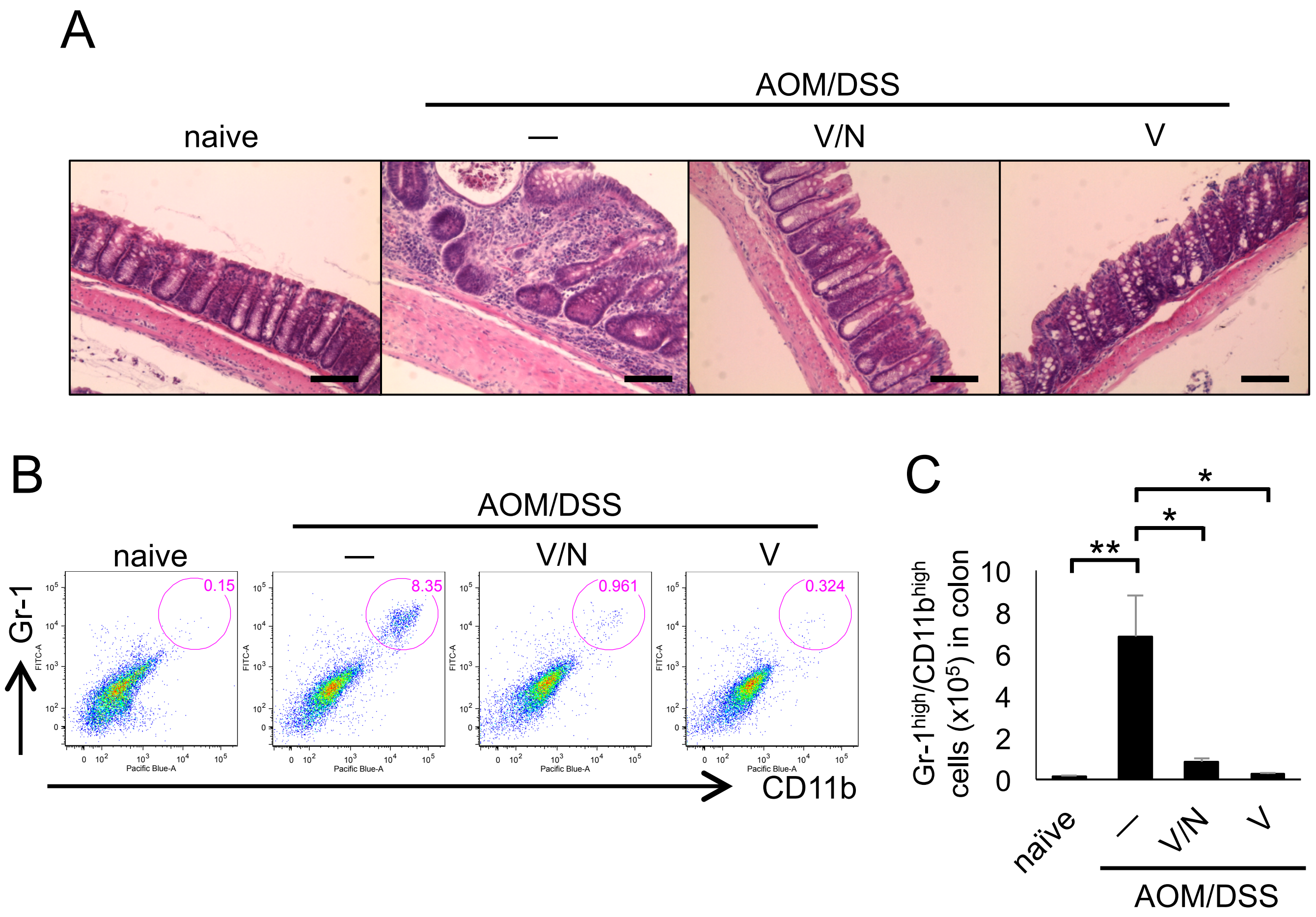

Supplementary figure S4. Colitis severity and myeloid infiltration into colon at day 24

A. H&E staining of distal colon at day 24. Scale bar: 100 μm.

B. Flow cytometric analysis of immune cells in lamina propria of colon at day 24. Single cell suspensions were stained with FITC-conjugated anti-Gr-1, eFluor450-conjugated anti-CD11b, and analyzed on a FACS Canto flow cytometer as described in the Materials and Methods.

C. Statistical analysis for flow cytometry.

V/N, vancomycin and neomycin; V, vancomycin. Data represent mean ± SEM (n = 4). *$p < 0.05$, **$p < 0.01$, ***$p < 0.001$.

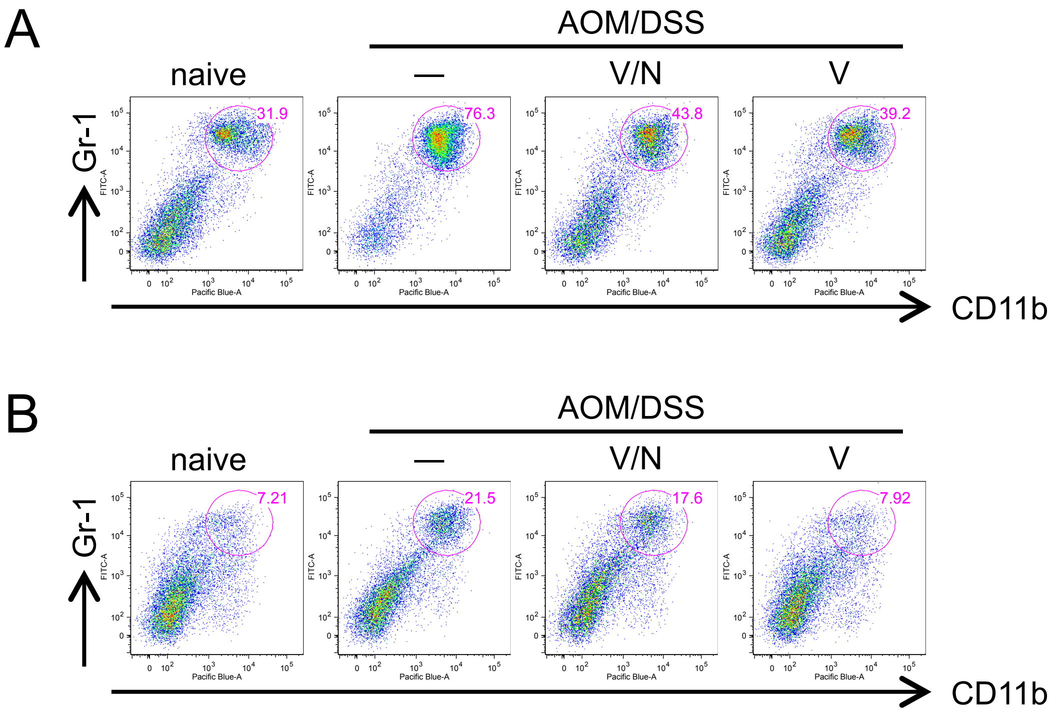

Supplementary figure S5. Enhancement of Gr-1^{high}/CD11b^{high} neutrophils in bone marrow and spleen by colitis

A, B. Flow cytometric analysis of immune cells in bone marrow (A) and spleen (B) from AOM/DSS-treated mice at day 24. Single cell suspensions were stained with FITC-conjugated anti-Gr-1, eFluor450-conjugated anti-CD11b, and analyzed on a FACS Canto flow cytometer as described in the Materials and Methods. Data are representative of three separate experiments.

V/N, vancomycin and neomycin; V, vancomycin.

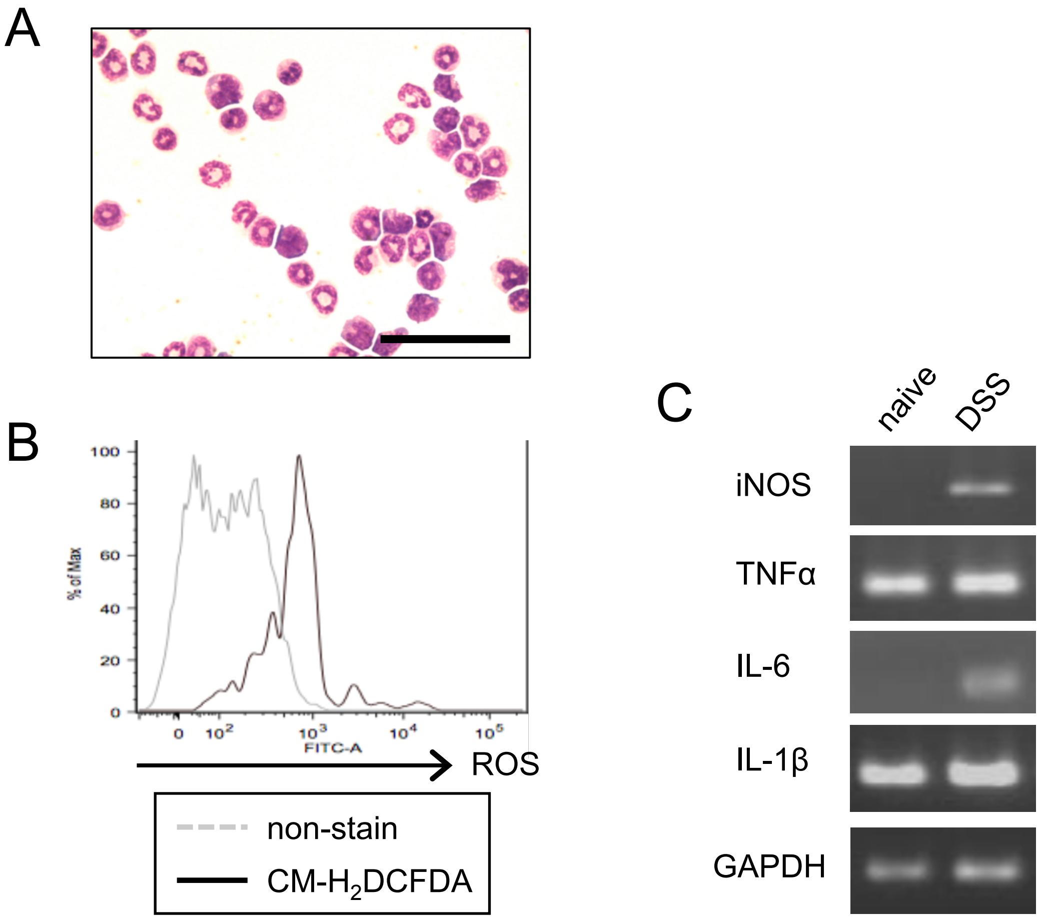

Supplementary figure S6. Characteristics of Gr-1^{high}/CD11b^{high} cells

A. May-Grunwald Giemsa stained Gr-1^{high}/CD11b^{high} cells from bone marrow of colitis mice (at day 12). Scale bar: 50 μm.

B. ROS production from Gr-1^{high}/CD11b^{high} cells in DSS-treated colon (at day 12).

C. RT-PCR analysis of sorted Gr-1^{high}/CD11b^{high} cells from bone marrow of colitis mice (at day 12). Data are representative of three separate experiments. The original blots are presented in Supplementary figure S8.

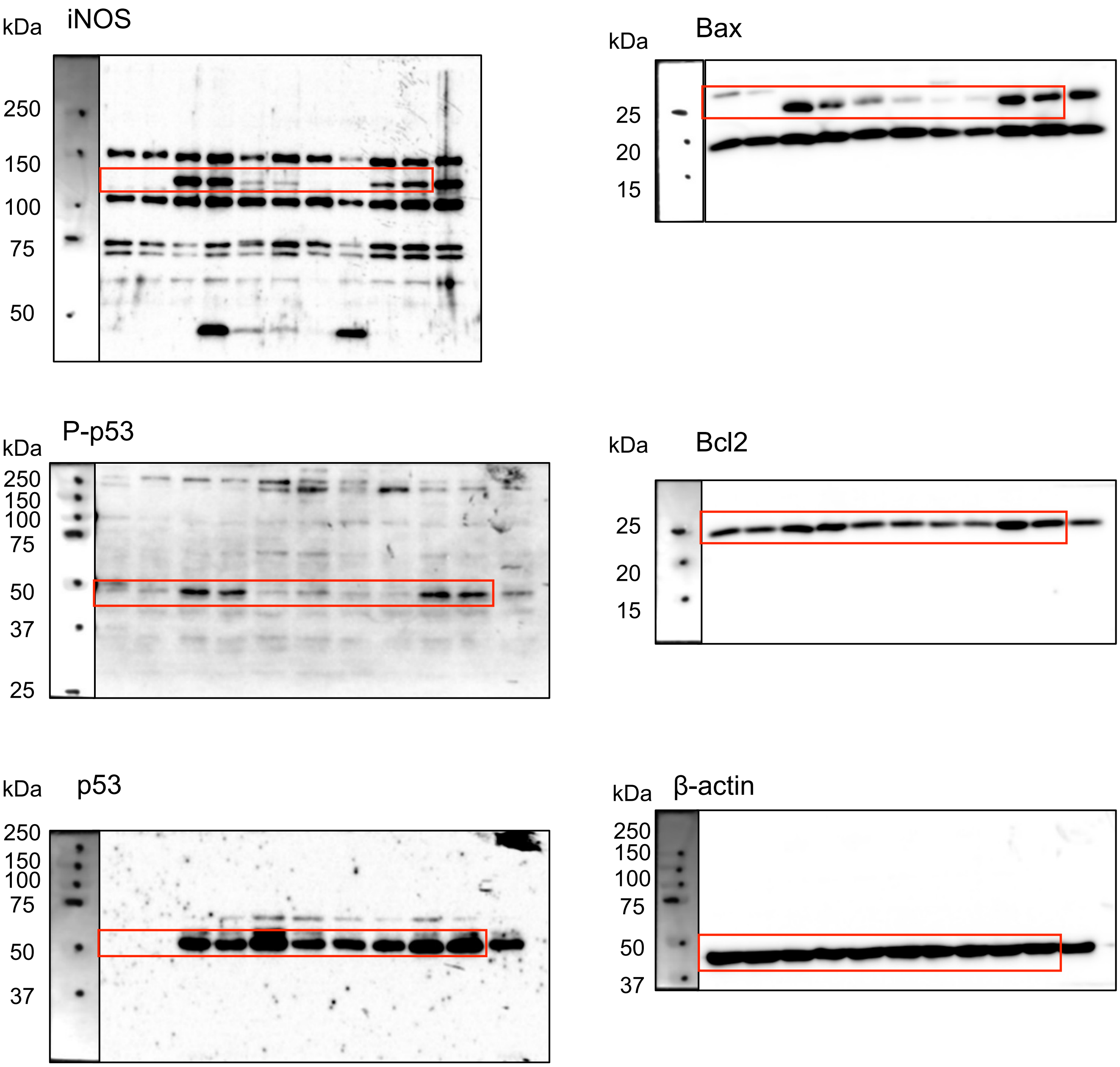

Supplementary figure S7. Full length blot for Figure 2C.

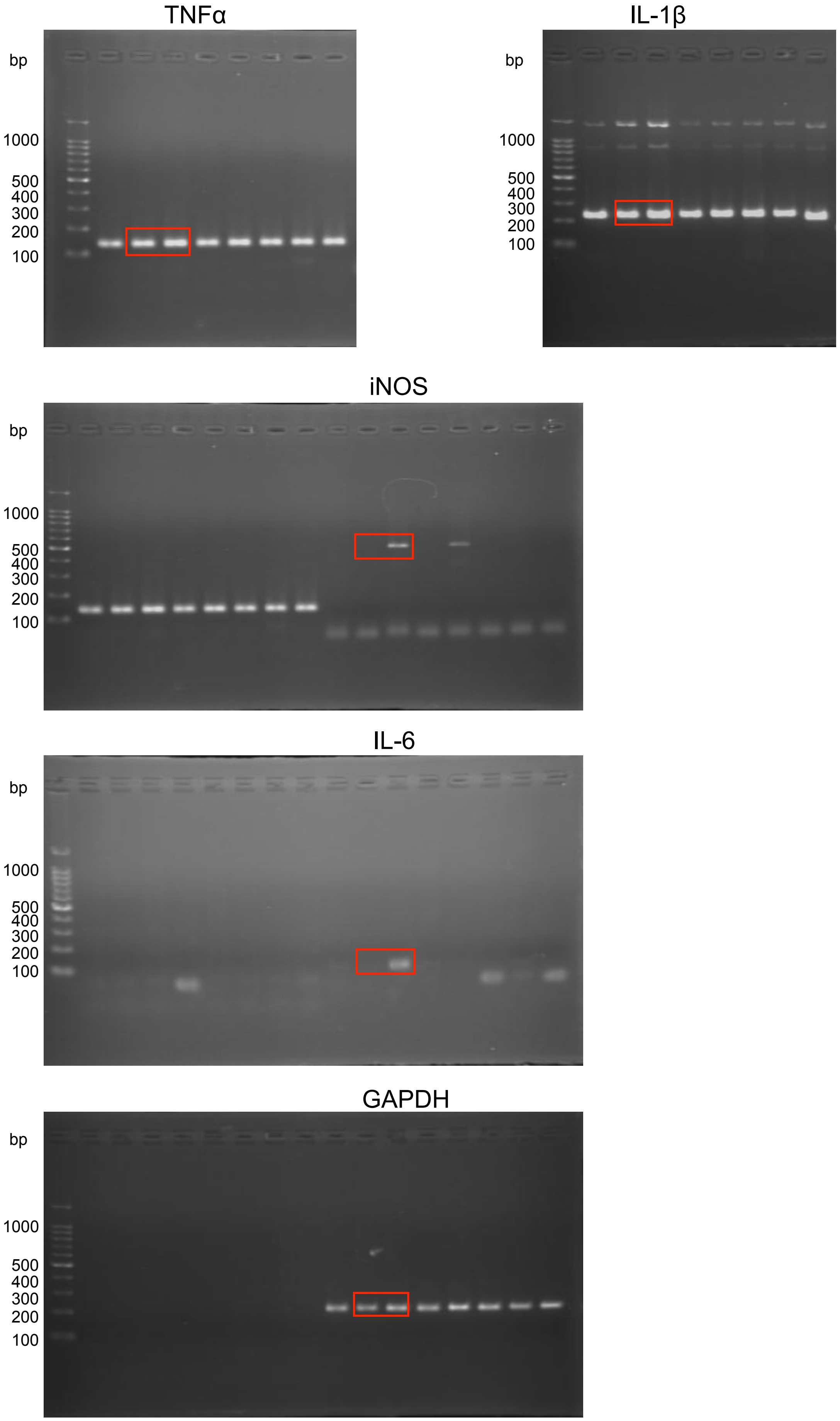

Supplementary figure S8. Full length blot for Supplementary figure 6C.
